# Assessing the Impact of Continuous Vaccination and Voluntary Isolation on the Dynamics of COVID-19: A Mathematical Optimal Control of SEIR Epidemic Model

**DOI:** 10.1155/2022/3309420

**Published:** 2022-06-02

**Authors:** Yue Yu, Manman Shi, Manfeng Hu, Jingxiang Zhang

**Affiliations:** School of Science, Jiangnan University, 1800 Lihu Avenue, Wuxi, Jiangsu 214122, China

## Abstract

In order to study the impact of continuous vaccination and voluntary isolation for the COVID-19, a susceptible-exposed-infected-recovered-quarantine-vaccines (SEIR-QV) model is proposed. A basic regeneration number *R*_0_ is defined to determine the extinction or persistence of the disease. We numerically analyze the impact of key parameters based on actual parameters of COVID-19, such as the vaccination rate, population importation rate, and natural (or causal) mortality transmission rate on the dynamics of disease transmission. Then we obtain sensitivity indices of some parameters on *R*_0_ by sensitivity analysis. Finally, the stability of the system and the effectiveness of the optimal control strategy are verified by numerical simulation.

## 1. Introduction

Since the outbreak of the coronavirus disease 2019 (COVID-19), it has been significantly impacting the world economy and our lives. Globally, as of 4:00 pm CET, 28 February 2022, there have been 434,154,739 confirmed cases of COVID-19, including 5,944,342 deaths, reported to the WHO. As of 26 February 2022, a total of 10,585,766,316 vaccine doses have been administered.

With the emergence of multiple mutant strains of novel coronavirus, including Alpha, Beta, Gamma, Delta, and Omicron, many scholars further explore the transmission and epidemic pattern of COVID-19 from different view. These research results are helpful to learn about the transmission and mode of infectious diseases and provide reliable information for the prediction and control of infectious diseases.

In the study of the dynamics of infectious diseases, the susceptibility-infection-recovery (SIR) model was mainly established by Kermack and McKendrick in 1927 with the kinetic method. In order to better study the characteristics of infectious diseases and obtain the best prevention strategies, scholars propose many improved models based on the traditional SIR model for different situations. By introducing time variation factors, Chen et al. [1] present a new SIR model, which has stronger adaptability and robustness in predicting the number of confirmed cases and inflection points of infectious diseases. Wang et al. [2] propose a SIR model with time-varying isolation protocol. In this model, the dynamic evolution of infectious diseases can be comprehensively analyzed and evaluated. In addition, Hota et al. [3] introduce a closed-loop framework combined with the SIR model and expound the significance of early detection through two feasible optimization questions.

In view of the latent characteristics of some infectious diseases, SEIR models are further proposed based on the SIR model. Li et al. [4] fit the basic regeneration number curve based on the SEIR model. Trends in COVID-19 outbreaks in China, the USA, India, and Iran are predicted and analyzed. The results show that the rapid and efficient isolation measures adopted in China are significant in suppressing COVID-19. In addition, some new models take into account the influence of different factors on infectious diseases, such as the presence of temperature and humidity [5, 6]. It is concluded that COVID-19 is more infectious and lethal at low temperatures and humidity. Besides, many research studies show that the different epidemic prevention measures, such as mandatory isolation, wearing masks, vaccination, and government control policies, have significant effects on preventing the rapid spread of COVID-19 [7–9].

Due to the problem of asymptomatic infected persons in infectious diseases, Yu et al. [10] propose an SEIR-AQ model by considering factors including prevention and control efforts, isolation strategies, and asymptomatic infected persons. The SEIR-AQ model can well anticipate the spread trend of COVID-19, providing technical support for the scientific assessment of the infectious disease situation. Analysis from the SEIR-AQ model shows that prevention and control isolation, medical follow-up isolation, and other measures have a significant inhibitory impact on the spread of COVID-19. In addition, some scholars use deep learning methods to study the impact of the COVID-19 outbreak on human society [11]. Some other scholars have analyzed and studied the transmission trends of the COVID-19 outbreak. The spatiotemporal evolution and transmission trends of the COVID-19 epidemic are analyzed using an ontological modeling approach by Liu et al. [12].

Although control measures, vaccination, latent infectivity, and other factors are considered in these studies, the stability and existence of the model are not discussed from the mathematical view point. Therefore, we establish a new susceptible-exposed-infected-recovered-quarantine-vaccines (SEIR-QV) model based on the latent period and the influence of prevention and control measures of COVID-19. In mathematical theory, the extinction or persistence of a disease can be determined based on the basic reproduction number and the Lyapunov function. We numerically simulate and analyze the impact of key real-world parameters of COVID-19. What's more, our sensitivity analysis indicates that the related parameters have significant effects on the stability and existence of the SEIR-QV model.

## 2. The SEIR-QV Model

In this paper, the susceptible-exposed-infectious-recovered-asymptomatic-quarantine-vaccines (SEIR-AQV) model is developed based on the traditional SEIR model. The SEIR-AQV model takes into account the effect of isolation based on the traditional model and divides the population into unisolated susceptible people (*S*), unisolated exposed people (*E*), unisolated infected people (*I*), isolated susceptible people (*S*_*q*_), isolated exposed people (*E*_*q*_), asymptomatic infected people (*A*), hospitalized people (H), and recovered people (*R*).

The SEIR-AQV model has the following assumptions: first assumption: medical resources are adequate, i.e., isolated exposed people can be directly converted to hospitalized people after diagnosis; second assumption: once infected with COVID-19, one must undergo inpatient treatment to recover, i.e., infected people are unlikely to recover on their own; third assumption: both isolated exposed people and hospitalized people are isolated from the outside world, i.e., they are not infectious; fourth assumption: the infected people will be immune after recovery, i.e., he or she will not become susceptible again after recovery.

**Figure 1:**
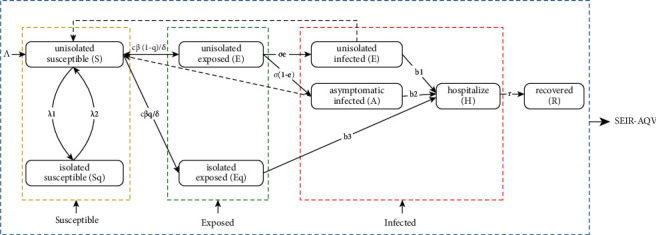
Bin view of the population transformation relationship of the SEIR-AQV model.

The bin transformation relationship is shown in Figure 1.

The equations of the SEIR-AQV model are as follows:

(1) $$\begin{array}{c} \left\{\begin{array}{c} \frac{\text{d}S}{\text{d}t}=\Lambda -\frac{c\beta }{\delta }S\left(I+\theta A+\upsilon E\right)+\lambda_{2}S_{q}-\left(\lambda_{1}+\eta \right)S,\\ \frac{\text{d}S_{q}}{\text{d}t}=\lambda_{1}S-\left(\lambda_{2}+\eta \right)S_{q},\\ \frac{\text{d}E}{\text{d}t}=\frac{c\left(1-q\right)\beta }{\delta }S\left(I+\theta A+\upsilon E\right)-\left(\sigma +\eta \right)E,\\ \frac{\text{d}E_{q}}{\text{d}t}=\frac{cq\beta }{\delta }S\left(I+\theta A+\upsilon E\right)-\left(b_{3}+\eta \right)E_{q},\\ \frac{\text{d}I}{\text{d}t}=\sigma eE-\left(b_{1}+\alpha_{1}+\eta \right)I,\\ \frac{\text{d}A}{\text{d}t}=\sigma \left(1-e\right)E-\left(b_{2}+\alpha_{2}+\eta \right)A,\\ \frac{\text{d}H}{\text{d}t}=b_{1}I+b_{2}A+b_{3}E_{q}-\left(r+\alpha_{3}+\eta \right)H,\\ \frac{\text{d}R}{\text{d}t}=rH-\eta R, \end{array}\right. \end{array}$$

where *c* means the rate of exposure; *η* means the natural mortality rate; *q* represents the proportion of isolated; *δ* represents the vaccine coverage rate; *β* represents the probability of transmission; *θ* stands for the ratio of *A* relative to the transmission capacity of *I*; *υ* stands for the ratio of *E* relative to the transmission capacity of *I*; *b*_1_, *b*_2_, *b*_3_ stand for the conversion of *I*, *A*, and*E*_*q*_, respectively to *H*; *λ*_1_ represent the rate of conversion of *S* into *S*_*q*_; *λ*_2_ stands for the rate of conversion of *S*_*q*_ into *S*; *σ* stands for the ratio of *E* to *I* conversion and *σ*=1/Incubation period; *e* means the ratio of *E* to *I* conversion; *α*_1_, *α*_2_, *α*_3_ mean the rate of conversion of *I*, *A*, and the rate of cause-specific death of *H*; *r* means the rate of recovery of *H*; and Λ means the rate of population importation.

**Figure 2:**
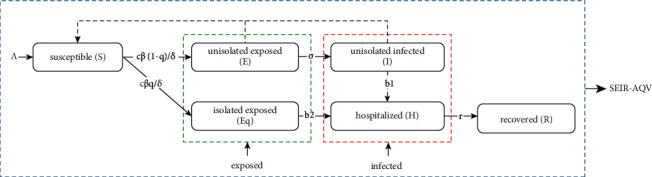
Bin view of the population transformation relationship of the SEIR-QV model.

However, given the reality of the COVID-19 epidemic, the status of isolated susceptible people is unlikely to persist over time, and isolated susceptible people have no translational relationship to hamlets other than unisolated susceptible people. Therefore, the population type of isolated susceptible people and the parameters involved in it are removed. Considering also that the proportion of asymptomatic infected people is very small and has minimal effect on the overall transmission trend of the COVID-19 outbreak, the population type of asymptomatic infected people and its involved parameters are also removed. Therefore, we improved the SEIR-AQV model in the context of the COVID-19 outbreak. We construct a new seasonal susceptible-exposed-infected-removed-quarantine-vaccines (SEIR-QV) model with the population transformation relationship bin view shown in Figure 2.

The equations of the SEIR-QV model are as follows:

(2) $$\begin{array}{c} \left\{\begin{array}{c} \frac{\text{d}S}{\text{d}t}=\Lambda -\frac{c\beta }{\delta }S\left(I+\upsilon E\right)-\eta S,\\ \frac{\text{d}E}{\text{d}t}=\frac{c\left(1-q\right)\beta }{\delta }S\left(I+\upsilon E\right)-\left(\sigma +\eta \right)E,\\ \frac{\text{d}E_{q}}{\text{d}t}=\frac{cq\beta }{\delta }S\left(I+\upsilon E\right)-\left(b_{2}+\eta \right)E_{q},\\ \frac{\text{d}I}{\text{d}t}=\sigma E-\left(b_{1}+\alpha_{1}+\eta \right)I,\\ \frac{\text{d}H}{\text{d}t}=b_{1}I+b_{2}E_{q}-\left(r+\alpha_{2}+\eta \right)H,\\ \frac{\text{d}R}{\text{d}t}=rH-\eta R, \end{array}\right. \end{array}$$

where *α*_1_, *α*_2_ represent the rate of cause-specific death of unisolated infected *I* and hospitalized *H*, respectively, and *b*_1_, *b*_2_ represent the rate of conversion of unisolated infected *I* and isolated exposed *E*_*q*_ to hospitalized *H*, respectively.

### 2.1. Proof of Stability of the SEIR-QV Model

To study the stability of equation (2), it is sufficient to study the stability of the following three formulas in (2) [13, 14]:

(3) $$\begin{array}{c} \left\{\begin{array}{c} \frac{\text{d}S}{\text{d}t}=\Lambda -\frac{c\beta }{\delta }S\left(I+\upsilon E\right)-\eta S,\\ \frac{\text{d}E}{\text{d}t}=\frac{c\left(1-q\right)\beta }{\delta }S\left(I+\upsilon E\right)-\left(\sigma +\eta \right)E,\\ \frac{\text{d}I}{\text{d}t}=\sigma E-\left(b_{1}+\alpha_{1}+\eta \right)I. \end{array}\right. \end{array}$$

Considering the biological significance of the system, the dynamical properties of equation (3) are discussed mainly in the closed set Ω={(*S*, *E*, *I*)∈*R*_+_^3^*S|* ≥ 0,  *E* ≥ 0,  *I* ≥ 0}, where *R*_+_^3^ denotes the first trigonometric limit of *R*^3^ and contains the boundary.

#### 2.1.1. Existence of Equilibrium Point

The equilibrium point of (3) satisfies

(4) $$\begin{array}{c} \left\{\begin{array}{c} \frac{\text{d}S}{\text{d}t}=0,\\ \frac{\text{d}E}{\text{d}t}=0,\\ \frac{\text{d}I}{\text{d}t}=0. \end{array}\right. \end{array}$$

When *I*=0, (3) has the point of diseased equilibrium *P*^0^=(*S*^0^, *E*^0^, *I*^0^)=(Λ/*η*, 0,0).

When *I* ≠ 0, from d*I*/d*t*=0, we get *I*=*σE*/*α*_1_+*b*_1_+*η*; from d*E*/d*t*=0, we get *S*(*I*+*υE*)=*δ*(*σ*+*η*)*E*/*cβ*(1 − *q*), from dS/d*t*=0, we get *S*(*I*+*υE*)=(*δ*/*cβ*)(Λ − *ηS*), so *S*=(Λ/*η*) − ((*σ*+*η*)*E*/(1 − *q*)*η*).

Bring *I*=*σE*/*α*_1_+*b*_1_+*η* and *S*=(Λ/*η*) − ((*σ*+*η*)*E*/(1 − *q*)*η*) into *S*(*I*+*υE*)=*δ*/*cβ*(Λ − *ηS*) to obtain the endemic equilibrium point *P*^*∗*^=(*S*^*∗*^, *E*^*∗*^, *I*^*∗*^), where

(5) $$\begin{array}{c} S^{*}=\frac{\delta \left(\sigma +\eta \right)\left(\alpha_{1}+b_{1}+\eta \right)}{c\beta \left(1-q\right)\left[\sigma +\upsilon \left(\alpha_{1}+b_{1}+\eta \right)\right]},\\ E^{*}=\frac{\Lambda \left(1-q\right)}{\sigma +\eta }-\frac{\delta \eta \left(\alpha_{1}+b_{1}+\eta \right)}{c\beta \left[\sigma +\upsilon \left(\alpha_{1}+b_{1}+\eta \right)\right]},\\ I^{*}=\frac{\sigma \Lambda \left(1-q\right)}{\left(\sigma +\eta \right)\left(\alpha_{1}+b_{1}+\eta \right)}-\frac{\sigma \delta \eta }{c\beta \left[\sigma +\upsilon \left(\alpha_{1}+b_{1}+\eta \right)\right]}. \end{array}$$

#### 2.1.2. Basic Regeneration Number

The basic regeneration number (*R*_0_) indicates the number of people infected by a patient during the average disease period when all are susceptible at the beginning of the disease. *R*_0_=1 can be used as a threshold to decide whether the disease is extinguished or not. When *R*_0_ < 1, the disease will become extinct. When *R*_0_ > 1, the disease will persist. The basic regeneration number is closely related to the stability of the endemic equilibrium point.

Next, we study the basic regeneration number *R*_0_ of equation (3). Let *x*=(*E*, *I*, *S*)^*T*^, then equation (3) can be rewritten as

(6) $$\begin{array}{c} \frac{\text{d}x}{\text{d}t}=F\left(x\right)-V\left(x\right), \end{array}$$

where

(7) $$\begin{array}{c} F\left(x\right)=\left(\begin{array}{c} \frac{c\left(1-q\right)\beta }{\delta }S\left(I+\upsilon E\right)\\ 0\\ 0 \end{array}\right),\\ V\left(x\right)=\left(\begin{array}{c} \left(\sigma +\eta \right)E\\ \left(\alpha_{1}+b_{1}+\eta \right)I-\sigma E\\ \eta S+\frac{c\beta }{\delta }S\left(I+\upsilon E\right)-\Lambda \end{array}\right). \end{array}$$

The Jacobi matrices of *F*(*x*) and *V*(*x*) at the disease-free equilibrium *P*^0^=((Λ/*η*), 0,0) are, respectively,

(8) $$\begin{array}{c} DF\left(P^{0}\right)=\left(\begin{array}{cc} F_{2\times 2} & 0\\ 0 & 0 \end{array}\right)\\ =\left(\begin{array}{ccc} \frac{c\left(1-q\right)\beta \upsilon \Lambda }{\delta \eta } & \frac{c\left(1-q\right)\beta \Lambda }{\delta \eta } & 0\\ 0 & 0 & 0\\ 0 & 0 & 0 \end{array}\right),\\ DV\left(P^{0}\right)=\left(\begin{array}{cc} V_{2\times 2} & 0\\ \begin{array}{cc} \frac{c\beta \upsilon \Lambda }{\delta \eta } & \frac{c\beta \Lambda }{\delta \eta } \end{array} & \eta \end{array}\right)\\ =\left(\begin{array}{ccc} \sigma +\eta & 0 & 0\\ -\sigma & \alpha_{1}+b_{1}+\eta & 0\\ \frac{c\beta \upsilon \Lambda }{\delta \eta } & \frac{c\beta \Lambda }{\delta \eta } & \eta \end{array}\right), \end{array}$$

where

(9) $$\begin{array}{c} F=\left(\begin{array}{cc} \frac{c\left(1-q\right)\beta \upsilon \Lambda }{\delta \eta } & \frac{c\left(1-q\right)\beta \Lambda }{\delta \eta }\\ 0 & 0 \end{array}\right),\\ V=\left(\begin{array}{cc} \sigma +\eta & 0\\ -\sigma & \alpha_{1}+b_{1}+\eta \end{array}\right). \end{array}$$

The basic regeneration number, denoted by *R*_0_, is thus given by the following equation:

(10) $$\begin{array}{c} R_{0}=\rho \left(FV^{-1}\right)\\ =\frac{c\Lambda \beta \left(1-q\right)}{\eta \delta \left(\eta +\sigma \right)}\left[\upsilon +\frac{\sigma }{\alpha_{1}+b_{1}+\eta }\right]. \end{array}$$

#### 2.1.3. Proof of Stability of the SEIR-QV Model

**Theorem 1 .**

For equation (3), the disease-free equilibrium *P*^0^=((Λ/*η*), 0,0) is locally asymptotically stable if *R*_0_ < 1.

**Proof**

Linearizing equation (3) at the disease-free equilibrium point *P*^0^=((Λ/*η*), 0,0), we obtain the linearization matrix at point *P*^0^=((Λ/*η*), 0,0) as the following equation:

(11) $$\begin{array}{c} J\left(P^{0}\right)=\left(\begin{array}{ccc} -\eta & -\frac{c\beta \upsilon \Lambda }{\delta \eta } & -\frac{c\beta \Lambda }{\delta \eta }\\ 0 & \frac{c\left(1-q\right)\beta \upsilon \Lambda }{\delta \eta }-\left(\sigma +\eta \right) & \frac{c\left(1-q\right)\beta \Lambda }{\delta \eta }\\ 0 & \sigma & -\left(\alpha_{1}+b_{1}+\eta \right) \end{array}\right). \end{array}$$

The characteristic equation of this matrix is det(*λL* − *J*(*P*^0^)), where *L* is a 3 × 3 unit matrix. Expanding it gives

(12) $$\begin{array}{c} \left(\lambda +\eta \right)\left(\lambda^{2}+\left(\left(\alpha_{1}+b_{1}+\eta \right)+\left(\sigma +\eta \right)-\frac{c\left(1-q\right)\beta \upsilon \Lambda }{\delta \eta }\right)\lambda +\left(\alpha_{1}+b_{1}+\eta \right)\left(\sigma +\eta \right)\left(1-R_{0}\right)\right)=0. \end{array}$$

Obviously, this characteristic equation has a negative characteristic root *λ*=−*η*. The other characteristic roots satisfy the following equation:

(13) $$\begin{array}{c} \lambda^{2}+\left(\left(\alpha_{1}+b_{1}+\eta \right)+\left(\sigma +\eta \right)-\frac{c\left(1-q\right)\beta \upsilon \Lambda }{\delta \eta }\right)\lambda +\left(\alpha_{1}+b_{1}+\eta \right)\left(\sigma +\eta \right)\left(1-R_{0}\right)=0. \end{array}$$

It is known that *R*_0_=*ρ*(*FV*^−1^)=(*c*Λ*β*(1 − *q*)/*ηδ*(*η*+*σ*))[*υ*+(*σ*/*α*_1_+*b*_1_+*η*)] ≤ 1.

Let *R*_1_=*R*_0_ − (*c*Λ*β*(1 − *q*)/*ηδ*(*η*+*σ*)) · (*σ*/*α*_1_+*b*_1_+*η*)=(*c*Λ*β*(1 − *q*)*υ*/*ηδ*(*η*+*σ*)) < 1, then

(14) $$\begin{array}{c} \left(\alpha_{1}+b_{1}+\eta \right)+\left(\sigma +\eta \right)-\frac{c\left(1-q\right)\beta \upsilon \Lambda }{\delta \eta }\\ =\left(\alpha_{1}+b_{1}+\eta \right)+\left(\sigma +\eta \right)\left[1-\frac{c\left(1-q\right)\beta \upsilon \Lambda }{\delta \eta \left(\sigma +\eta \right)}\right]\\ =\left(\alpha_{1}+b_{1}+\eta \right)+\left(\sigma +\eta \right)\left(1-R_{1}\right). \end{array}$$

So we can obtain

(15) $$\begin{array}{c} \lambda^{2}+\left(\left(\alpha_{1}+b_{1}+\eta \right)+\left(\sigma +\eta \right)\left(1-R_{1}\right)\right)\lambda +\left(\alpha_{1}+b_{1}+\eta \right)\left(\sigma +\eta \right)\left(1-R_{0}\right)=0. \end{array}$$

It is easy to verify for *R*_0_ < 1 and *R*_1_ < 1

(16) $$\begin{array}{c} \left(\left(\alpha_{1}+b_{1}+\eta \right)+\left(\sigma +\eta \right)\left(1-R_{1}\right)\right)\left(\alpha_{1}+b_{1}+\eta \right)\left(\sigma +\eta \right)\left(1-R_{0}\right)>0, \end{array}$$

that the roots of the quadratic equation are as follows:

(17) $$\begin{array}{c} \lambda^{2}+\left(\left(\alpha_{1}+b_{1}+\eta \right)+\left(\sigma +\eta \right)\left(1-R_{1}\right)\right)\lambda +\left(\alpha_{1}+b_{1}+\eta \right)\left(\sigma +\eta \right)\left(1-R_{0}\right)=0. \end{array}$$

All have negative real parts, i.e., the disease-free equilibrium point *P*^0^=((Λ/*η*), 0,0) of equation (3) is proved to be locally asymptotically stable.

**Theorem 2 .**

For equation (3), the disease-free equilibrium *P*^0^=((Λ/*η*), 0,0) is globally asymptotically stable if *R*_0_ ≤ 1.

**Proof**

By considering the Lyapunov function,

(18) $$\begin{array}{c} V\left(t\right)=x_{1}E\left(t\right)+x_{2}I\left(t\right). \end{array}$$

Clearly, the solution of *V*(*t*) along equation (3) has *V*(*t*) ≥ 0 and *V*(*t*)=0 when and only when *E*(*t*)=0 and *I*(*t*)=0. The full derivative of the solution of the function *V*(*t*) along equation (3) is as follows:

(19) $$\begin{array}{c} \frac{\text{d}V\left(t\right)}{\text{d}t}=x_{1}\frac{\text{d}E\left(t\right)}{\text{d}t}+x_{2}\frac{\text{d}I\left(t\right)}{\text{d}t}\\ =x_{1}\left[\frac{c\left(1-q\right)\beta }{\delta }S\left(I+\upsilon E\right)-\left(\sigma +\eta \right)E\right]+x_{2}\left[\sigma E-\left(b_{1}+\alpha_{1}+\eta \right)I\right]\\ =x_{1}\frac{c\left(1-q\right)\beta }{\delta }SI+\left[\frac{x_{1}c\left(1-q\right)\beta \upsilon }{\delta }S-x_{1}\left(\sigma +\eta \right)+x_{2}\sigma \right]E-x_{2}\left(b_{1}+\alpha_{1}+\eta \right)I\\ \leq \frac{c\left(1-q\right)\beta \Lambda }{\delta \eta }x_{1}I-\left(b_{1}+\alpha_{1}+\eta \right)x_{2}I+\left[\frac{c\left(1-q\right)\beta \upsilon \Lambda }{\delta \eta }x_{1}-\left(\sigma +\eta \right)x_{1}+\sigma x_{2}\right]E. \end{array}$$

Take *x*_1_=*σ*, *x*_2_=*σ*+*η*(*c*(1 − *q*)*βυ*Λ/*δη*).

Then,

(20) $$\begin{array}{c} \frac{\text{d}V\left(t\right)}{\text{d}t}\leq \frac{c\left(1-q\right)\beta \Lambda \sigma }{\delta \eta }I-\left(b_{1}+\alpha_{1}+\eta \right)\left[\left(\sigma +\eta \right)-\frac{c\left(1-q\right)\beta \upsilon \Lambda }{\delta \eta }\right]I\\ =\left\{\frac{c\left(1-q\right)\beta \Lambda \sigma }{\delta \eta }+\left(b_{1}+\alpha_{1}+\eta \right)\frac{c\left(1-q\right)\beta \upsilon \Lambda }{\delta \eta }-\left(b_{1}+\alpha_{1}+\eta \right)\left(\sigma +\eta \right)\right\}I\\ =\left\{\frac{c\left(1-q\right)\beta \Lambda }{\delta \eta }\left[\sigma +\left(b_{1}+\alpha_{1}+\eta \right)\upsilon \right]-\left(b_{1}+\alpha_{1}+\eta \right)\left(\sigma +\eta \right)\right\}I\\ =\left\{\left(b_{1}+\alpha_{1}+\eta \right)\left(\sigma +\eta \right)\frac{c\left(1-q\right)\beta \Lambda }{\delta \eta \left(\sigma +\eta \right)}\left[\upsilon +\frac{\sigma }{b_{1}+\alpha_{1}+\eta }\right]-\left(b_{1}+\alpha_{1}+\eta \right)\left(\sigma +\eta \right)\right\}I,\\ =\left(b_{1}+\alpha_{1}+\eta \right)\left(\sigma +\eta \right)\left(R_{0}-1\right)I, \end{array}$$

where the inequality sign is obtained based on *S* ≤ *S*^0^. When *R*_0_ ≤ 1, there is d*V*(*t*)/d*t* ≤ 0. Thus, d*V*(*t*)/d*t* ≤ 0 when and only when *I*(*t*)=0. Therefore, if *R*_0_ ≤ 1, the maximum tight invariant set {(*S*, *E*, *I*) ∈ Ω*|*(d*V*(*t*)/d*t*)=0} is the single point set {*P*^0^}. Therefore, if *R*_0_ ≤ 1, the disease-free equilibrium point *P*^0^ of equation (3) is globally asymptotically stable in Ω.

**Theorem 3 .**

*S* ^*∗*^, *E*^*∗*^, *I*^*∗*^ are positive when *R*_0_ > 1.

**Proof**

(21) $$\begin{array}{c} S^{*}=\frac{\delta \left(\sigma +\eta \right)\left(\alpha_{1}+b_{1}+\eta \right)}{c\beta \left(1-q\right)\left[\sigma +\upsilon \left(\alpha_{1}+b_{1}+\eta \right)\right]}. \end{array}$$

It is known that the parameters are positive and *q* < 1, so *S*^*∗*^ > 0 obviously holds the following equation:

(22) $$\begin{array}{c} E^{*}=\frac{\Lambda \left(1-q\right)}{\left(\sigma +\eta \right)}-\frac{\delta \eta \left(\alpha_{1}+b_{1}+\eta \right)}{c\beta \left[\sigma +\upsilon \left(\alpha_{1}+b_{1}+\eta \right)\right]}\\ =\frac{c\beta \Lambda \left(1-q\right)\left[\sigma +\upsilon \left(\alpha_{1}+b_{1}+\eta \right)\right]-\delta \eta \left(\alpha_{1}+b_{1}+\eta \right)\left(\sigma +\eta \right)}{c\beta \left(\sigma +\eta \right)\left[\sigma +\upsilon \left(\alpha_{1}+b_{1}+\eta \right)\right]}\\ =\frac{\left(c\beta \Lambda \left(1-q\right)/\left(\sigma +\eta \right)\right)\left[\sigma +\upsilon \left(\alpha_{1}+b_{1}+\eta \right)\right]-\delta \eta \left(\alpha_{1}+b_{1}+\eta \right)}{c\beta \left[\sigma +\upsilon \left(\alpha_{1}+b_{1}+\eta \right)\right]}\\ =\frac{\left(c\beta \Lambda \left(1-q\right)/\delta \eta \left(\sigma +\eta \right)\right)\left[\sigma +\upsilon \left(\alpha_{1}+b_{1}+\eta \right)\right]-\left(\alpha_{1}+b_{1}+\eta \right)}{\left(c\beta /\delta \eta \right)\left[\sigma +\upsilon \left(\alpha_{1}+b_{1}+\eta \right)\right]}\\ =\frac{\left(\alpha_{1}+b_{1}+\eta \right)\left\{\left(c\beta \Lambda \left(1-q\right)/\delta \eta \left(\sigma +\eta \right)\right)\left[\upsilon +\left(\sigma /\left(\alpha_{1}+b_{1}+\eta \right)\right)\right]-1\right\}}{\left(c\beta /\delta \eta \right)\left[\sigma +\upsilon \left(\alpha_{1}+b_{1}+\eta \right)\right]}\\ =\frac{\left(\alpha_{1}+b_{1}+\eta \right)\left(R_{0}-1\right)}{\left(c\beta /\delta \eta \right)\left[\sigma +\upsilon \left(\alpha_{1}+b_{1}+\eta \right)\right]}. \end{array}$$

It is known that *R*_0_ > 1, *q* < 1 and the parameters are positive, so *E*^*∗*^ > 0.

(23) $$\begin{array}{c} I^{*}=\frac{\sigma \Lambda \left(1-q\right)}{\left(\sigma +\eta \right)\left(\alpha_{1}+b_{1}+\eta \right)}-\frac{\sigma \delta \eta }{c\beta \left[\sigma +\upsilon \left(\alpha_{1}+b_{1}+\eta \right)\right]}\\ =\sigma \cdot \frac{c\beta \Lambda \left(1-q\right)\left[\sigma +\upsilon \left(\alpha_{1}+b_{1}+\eta \right)\right]-\delta \eta \left(\sigma +\eta \right)\left(\alpha_{1}+b_{1}+\eta \right)}{c\beta \left(\sigma +\eta \right)\left(\alpha_{1}+b_{1}+\eta \right)\left[\sigma +\upsilon \left(\alpha_{1}+b_{1}+\eta \right)\right]}\\ =\sigma \cdot \frac{\left(c\beta \Lambda \left(1-q\right)/\delta \eta \right)\left[\sigma +\upsilon \left(\alpha_{1}+b_{1}+\eta \right)\right]-\left(\sigma +\eta \right)\left(\alpha_{1}+b_{1}+\eta \right)}{\left(c\beta /\delta \eta \right)\left(\sigma +\eta \right)\left(\alpha_{1}+b_{1}+\eta \right)\left[\sigma +\upsilon \left(\alpha_{1}+b_{1}+\eta \right)\right]}\\ =\sigma \cdot \frac{\left(c\beta \Lambda \left(1-q\right)/\delta \eta \left(\sigma +\eta \right)\right)\left(\alpha_{1}+b_{1}+\eta \right)\left[\upsilon +\left(\sigma /\left(\alpha_{1}+b_{1}+\eta \right)\right)\right]-\left(\left(\alpha_{1}+b_{1}+\eta \right)\right)}{\left(c\beta /\delta \eta \right)\left(\alpha_{1}+b_{1}+\eta \right)\left[\sigma +\upsilon \left(\alpha_{1}+b_{1}+\eta \right)\right]}\\ =\sigma \cdot \frac{\left(c\beta \Lambda \left(1-q\right)/\delta \eta \left(\sigma +\eta \right)\right)\left[\upsilon +\left(\sigma /\left(\alpha_{1}+b_{1}+\eta \right)\right)\right]-1}{\left(c\beta /\delta \eta \right)\left[\sigma +\upsilon \left(\alpha_{1}+b_{1}+\eta \right)\right]}\\ =\sigma \cdot \frac{R_{0}-1}{\left(c\beta /\delta \eta \right)\left[\sigma +\upsilon \left(\alpha_{1}+b_{1}+\eta \right)\right]}. \end{array}$$

It is known that *R*_0_ > 1, *q* < 1 and the parameters are positive, so *I*^*∗*^ > 0.

**Theorem 4 .**

For equation (3), the endemic equilibrium *P*^*∗*^=(*S*^*∗*^, *E*^*∗*^, *I*^*∗*^) is locally asymptotically stable if *R*_0_ > 1.

**Proof**

Linearize equation (3) at the endemic equilibrium point *P*^*∗*^=(*S*^*∗*^, *E*^*∗*^, *I*^*∗*^) and obtain the linearization matrix at point *P*^*∗*^=(*S*^*∗*^, *E*^*∗*^, *I*^*∗*^) as follows:

(24) $$\begin{array}{c} J\left(P^{*}\right)=\left(\begin{array}{ccc} -\frac{c\beta }{\delta }\left(I^{*}+\upsilon E^{*}\right)-\eta & -\frac{c\beta \upsilon }{\delta }S^{*} & -\frac{c\beta }{\delta }S^{*}\\ \frac{c\left(1-q\right)\beta }{\delta }\left(I^{*}+\upsilon E^{*}\right) & \frac{c\left(1-q\right)\beta \upsilon }{\delta }S^{*}-\left(\sigma +\eta \right) & \frac{c\left(1-q\right)\beta }{\delta }S^{*}\\ 0 & \sigma & -\left(\alpha_{1}+b_{1}+\eta \right) \end{array}\right). \end{array}$$

The characteristic equation of this matrix is det(*λL* − *J*(*P*^*∗*^)), where *L* is a 3 × 3 unit matrix. Expanding it gives *λ*^3^+*Aλ*^2^+*Bλ*+*C*=0, where

(25) $$\begin{array}{c} A=-\frac{c\left(1-q\right)\beta \upsilon S^{*}}{\delta }+\left(\sigma +\eta \right)+\left(\alpha_{1}+b_{1}+\eta \right)+\eta +\frac{c\beta \left(I^{*}+\upsilon E^{*}\right)}{\delta },\\ B=-\frac{c\eta \left(1-q\right)\beta \upsilon S^{*}}{\delta }+\eta \left(\sigma +\eta \right)+\eta \left(\alpha_{1}+b_{1}+\eta \right)-\frac{c\sigma \left(1-q\right)\beta S^{*}}{\delta }\\ +\left(\alpha_{1}+b_{1}+\eta \right)\left[-\frac{c\left(1-q\right)\beta \upsilon S^{*}}{\delta }+\left(\sigma +\eta \right)\right]\\ +\frac{c\beta \left(\left(\alpha_{1}+b_{1}+\eta \right)+\left(\sigma +\eta \right)\right)\left(I^{*}+\upsilon E^{*}\right)}{\delta },\\ C=c\eta +\frac{c\beta \left(\alpha_{1}+b_{1}+\eta \right)\left(\sigma +\eta \right)\left(I^{*}+\upsilon E^{*}\right)}{\delta }. \end{array}$$

Then,

(26) $$\begin{array}{c} AB-C\geq \frac{c^{2}\beta^{2}{\left(1-q\right)}^{2}\upsilon \left[\upsilon \eta +\sigma +\upsilon \left(\alpha_{1}+b_{1}+\eta \right)\right]}{\delta^{2}}S^{{*}^{2}}+\left(f_{1}+f_{2}+\eta \right)\left[f_{1}f_{2}+\eta \left(f_{1}+f_{2}\right)\right]\\ -\frac{c^{2}\beta^{2}\left(1-q\right)\left[2\upsilon \left(\alpha_{1}+b_{1}+\eta \right)+2\upsilon \eta +\upsilon \sigma +\sigma \right]}{\delta^{2}}-\frac{c\beta f_{1}f_{2}}{\delta }-c\eta \\ -\frac{c\beta \left(1-q\right)\Lambda \left\{\upsilon f_{1}f_{2}+\upsilon \eta \left(f_{1}+f_{2}\right)+\left(\upsilon \eta +\sigma +\upsilon f_{1}\right)\left(f_{1}+f_{2}+\eta \right)\right\}}{\delta \eta }, \end{array}$$

where *f*_1_=*α*_1_+*b*_1_+*η* and *f*_2_=*σ*+*η*.

Due to *I*^*∗*^+*υE*^*∗*^ ≥ *I*^0^+*υE*^0^ and *S*^*∗*^ ≤ *S*^0^, *AB* − *C* > 0.

Therefore, the endemic equilibrium point *P*^*∗*^=(*S*^*∗*^, *E*^*∗*^, *I*^*∗*^) of equation (3) is locally asymptotically stable on Ω^0^.

**Theorem 5 .**

For equation (3), the endemic equilibrium *P*^*∗*^=(*S*^*∗*^, *E*^*∗*^, *I*^*∗*^) is globally asymptotically stable if *R*_0_ > 1.

**Proof**

Considering the Lyapunov function, we can obtain

(27) $$\begin{array}{c} V\left(t\right)=S-S^{*}-S^{*}\ln\frac{S}{S^{*}}+x_{1}\left(E-E^{*}-E^{*}\ln\frac{E}{E^{*}}\right)+x_{2}\left(I-I^{*}-I^{*}\ln\frac{I}{I^{*}}\right). \end{array}$$

The full derivative of the solution of the function *V*(*t*) along equation (3) is as follows:

(28) $$\begin{array}{c} \frac{\text{d}V\left(t\right)}{\text{d}t}=\left(1-\frac{S^{*}}{S}\right)\frac{\text{d}S}{\text{d}t}+x_{1}\left(1-\frac{E^{*}}{E}\right)\frac{\text{d}E}{\text{d}t}+x_{2}\left(1-\frac{I^{*}}{I}\right)\frac{\text{d}I}{\text{d}t}\\ =\left(1-\frac{S^{*}}{S}\right)\left[\Lambda -\frac{c\beta }{\delta }S\left(I+\upsilon E\right)-\eta S\right]+x_{2}\left(1-\frac{I^{*}}{I}\right)\left[\sigma E-\left(b_{1}+\alpha_{1}+\eta \right)I\right]\\ +x_{1}\left(1-\frac{E^{*}}{E}\right)\left[\frac{c\left(1-q\right)\beta }{\delta }S\left(I+\upsilon E\right)-\left(\sigma +\eta \right)E\right]\\ =\left(I+\upsilon E\right)\left[\frac{c\beta S^{*}}{\delta }+x_{1}\frac{c\left(1-q\right)\beta S}{\delta }-\frac{c\beta S}{\delta }-x_{1}\frac{c\left(1-q\right)\beta E^{*}S}{\delta E}\right]+\Lambda -\eta S-\frac{\Lambda S^{*}}{S}\\ +\eta S^{*}-x_{1}\left(\sigma +\eta \right)E+x_{1}\left(\sigma +\eta \right)E^{*}+x_{2}\sigma E-x_{2}\left(b_{1}+\alpha_{1}+\eta \right)I-x_{2}\frac{\sigma EI^{*}}{I}+x_{2}\left(b_{1}+\alpha_{1}+\eta \right)I^{*}. \end{array}$$

Bringing in *I*^*∗*^=*σE*^*∗*^/*α*_1_+*b*_1_+*η*, *S*^*∗*^=(Λ/*η*) − ((*σ*+*η*)*E*^*∗*^/(1 − *q*)*η*) yields

(29) $$\begin{array}{c} \frac{\text{d}V\left(t\right)}{\text{d}t}=\left(I+\upsilon E\right)\left\{\frac{c\beta }{\delta }\left[\frac{\Lambda }{\eta }-\frac{\left(\sigma +\eta \right)E^{*}}{\left(1-q\right)\eta }\right]+x_{1}\frac{c\left(1-q\right)\beta S}{\delta }-\frac{c\beta S}{\delta }-x_{1}\frac{c\left(1-q\right)\beta E^{*}S}{\delta E}\right\}\\ +\Lambda -\eta S+\left[\frac{\Lambda }{\eta }-\frac{\left(\sigma +\eta \right)E^{*}}{\left(1-q\right)\eta }\right]\left(\eta -\frac{\Lambda }{S}\right)-x_{1}\left(\sigma +\eta \right)E+x_{1}\left(\sigma +\eta \right)E^{*}+x_{2}\sigma E\\ -x_{2}\left(b_{1}+\alpha_{1}+\eta \right)I-\frac{x_{2}\sigma^{2}EE^{*}}{I\left(b_{1}+\alpha_{1}+\eta \right)}+x_{2}\left(b_{1}+\alpha_{1}+\eta \right)\frac{\sigma E^{*}}{\left(b_{1}+\alpha_{1}+\eta \right)}\\ =\frac{c\beta }{\delta }\left(I+\upsilon E\right)\left\{S^{*}+\left[x_{1}\left(1-q\right)-1\right]S-x_{1}\frac{\left(1-q\right)E^{*}S}{E}\right\}-\frac{{\left(\Lambda -\eta S\right)}^{2}}{\eta S}\\ +\frac{\left(\Lambda -\eta S\right)\left(\sigma +\eta \right)E^{*}}{\eta S\left(1-q\right)}+\left[x_{2}\sigma -x_{1}\left(\sigma +\eta \right)\right]E+\left[x_{2}\sigma +x_{1}\left(\sigma +\eta \right)\right]E^{*}\\ -x_{2}\left(b_{1}+\alpha_{1}+\eta \right)I-\frac{x_{2}\sigma^{2}EE^{*}}{I\left(b_{1}+\alpha_{1}+\eta \right)}. \end{array}$$

Take *x*_1_=2/1 − q and *x*_2_=2(*σ*+*η*)/*σ*(1 − *q*).

Then,

(30) $$\begin{array}{c} \frac{\text{d}V\left(t\right)}{\text{d}t}=\frac{c\beta }{\delta }\left(I+\upsilon E\right)\left[S^{*}+S-\frac{2E^{*}S}{E}\right]-\frac{{\left(\Lambda -\eta S\right)}^{2}}{\eta S}+\frac{\left(\Lambda -\eta S\right)\left(\sigma +\eta \right)E^{*}}{\eta S\left(1-q\right)}\\ +\frac{4\left(\sigma +\eta \right)}{\left(1-q\right)}E^{*}-\frac{2\left(\sigma +\eta \right)\left(b_{1}+\alpha_{1}+\eta \right)I}{\sigma \left(1-q\right)}-\frac{2\sigma \left(\sigma +\eta \right)EE^{*}}{I\left(b_{1}+\alpha_{1}+\eta \right)\left(1-q\right)}\\ =\frac{c\beta }{\delta }\left(I+\upsilon E\right)\left[S^{*}+\left(1-\frac{2E^{*}}{E}\right)S\right]-\frac{\left(\Lambda -\eta S\right)}{S}\left[\frac{\left(\Lambda -\eta S\right)}{\eta }-\frac{\left(\sigma +\eta \right)E^{*}}{\eta \left(1-q\right)}\right]\\ +2\frac{2\sigma \left(b_{1}+\alpha_{1}+\eta \right)\left(\sigma +\eta \right)E^{*}I-\left(\sigma +\eta \right)\left(b_{1}+\alpha_{1}+\eta \right)I^{2}-\sigma^{2}\left(\sigma +\eta \right)EE^{*}}{\sigma \left(1-q\right)\left(b_{1}+\alpha_{1}+\eta \right)I}. \end{array}$$

Since Λ − *η*S < Λ and when *E* > (*α*_1_+*b*_1_+*η*), *σ*^2^*E*^2^ < (*σ*^2^*EE*^*∗*^/*α*_1_+*b*_1_+*η*).

At this time,

(31) $$\begin{array}{c} \frac{\text{d}V\left(t\right)}{\text{d}t}\geq \frac{c\beta }{\delta }\left(I+\upsilon E\right)\left[S^{*}+\left(1-\frac{2E^{*}}{E}\right)S\right]+\frac{\left(\eta S-\Lambda \right)}{S}S^{*}+2\left(\sigma +\eta \right)\frac{{\left(I-\sigma E\right)}^{2}}{\sigma \left(1-q\right)\text{I}}, \end{array}$$

and when *E* > 2*E*^*∗*^ and *S* > Λ/*η*, 1 − 2*E*^*∗*^/*E* > 0, and *ηS* − Λ > 0 holds, so d*V*(*t*)/d*t* ≥ 0. Therefore, if *R*_0_ > 1, the endemic equilibrium point *P*^*∗*^=(*S*^*∗*^, *E*^*∗*^, *I*^*∗*^) of equation (3) is globally asymptotically stable on Ω^0^.

## 3. Numerical Experiments

### 3.1. Numerical Simulation

The stability of disease-free and endemic equilibrium points is discussed in this section, and this section uses the software Matlab to numerically simulate equation (3) to verify the above conclusions.

The initial values of the system are (0.7,0.2,0.1), and the parameters are assigned as *c*=2, *η*=0.00714, *β*=0.1, *υ*=1, *b*_1_=0.8, *σ*=1/7, *α*_1_=0.04, and Λ=0.00334, where *q* and *δ* are two variables. In this paper, we will take some random values to simulate the change of *S*, *E*, and *I* ratio when *R*_0_ < 1 and *R*_0_ > 1, respectively. When *q*=0.9, *δ* takes a random value between 0 and 1 so that *R*_0_ < 1 holds. When *δ*=1, *q* takes a random value between 0 and 1 so that *R*_0_ < 1 holds. Its value of *R*_0_ is less than 1 (*R*_0_ < 1) when q and delta take the values in Table 1. Its value of *R*_0_ is greater than 1 (*R*_0_ < 1) when *q* and delta take the values in Table 2.

**Figure 3:**
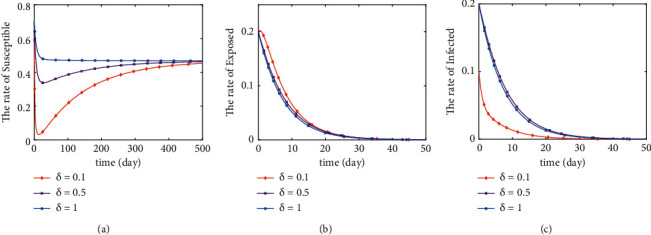
Stability of disease-free equilibrium point (*q*=0.9).

**Figure 4:**
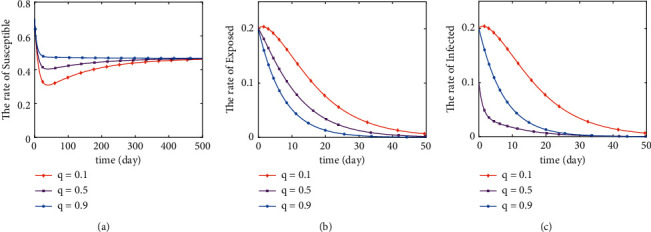
Stability of disease-free equilibrium point (*δ*=1).

**Figure 5:**
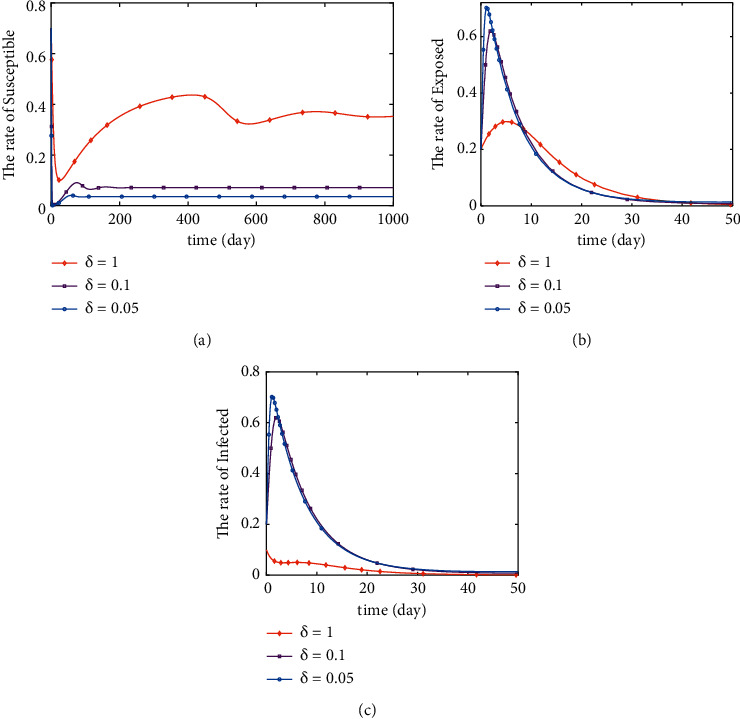
Stability of disease-free equilibrium point (*q*=0.1).

**Figure 6:**
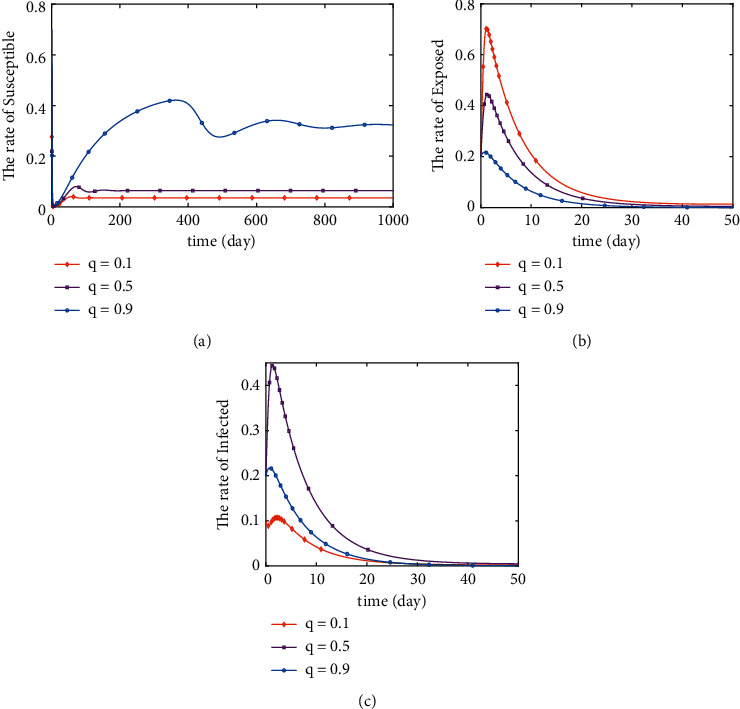
Stability of disease-free equilibrium point (*δ*=0.05).

The global asymptotic stability of both disease-free and endemic equilibrium points can be seen from Theorems 1–5. Figures 3–6 illustrates the correctness of the results obtained from the above theorem.

If *R*_0_ < 1, by numerically simulating the stability of the disease-free equilibrium point, we can analytically conclude the following points.  Figure 3 shows the variation curves of *S*, *E*, and *I* as the parameter *δ* varies when *q* is constant. The variation of *S* increases, and the rate of decrease and the rate of increase both increase with the increase of *δ*. The rate of decrease of *E* and *I* decreases with the increase of *δ*. Finally, *S*, *E*, and *I* all stabilized (see Figures 3(a)–3(c)). The change of *S* decreases, and the rate of decrease of *E* decreases with the increase of *q*. But the change of *I* has no obvious pattern with the increase of *q*. Finally, *S*, *E*, and *I* all tend to be stable (see Figures 4(a)–4(c)). Figures 3 and 4 illustrate that when *R*_0_ < 1, the changes of *q* and *δ* have significant effects on the trends of *S*, *E*, and *I*. And when *R*_0_ < 1, the disease-free equilibrium point is tending to be stable.

In Figures 5 and 6, the curve of *S*, *E*, and *I* variation with *q* and *δ* is simulated. By comparing the trend of *S*, *E*, and *I* with *q* in Figure 5 and the trend of *S*, *E*, and *I* with *δ* in Figure 6, we can draw the following conclusions. Increases in both *q* and *δ* lead to larger fluctuations in *S* and delayed stabilization times. It will lead to an increase in the value of *S* as it stabilizes. What is more, as *δ* increases, the rate of increase and decrease of *E* decreases. The monotonically increasing interval of *I* decreases and the rate of decrease of the monotonically decreasing interval decreases with the increase of *δ*. The maximum peak value of both *E* and *I* decreases with the increase of *δ*. And the time of reaching the maximum peak value of *E* is delayed and the time of reaching the maximum peak value of *I* is advanced. Furthermore, as *q* increases, the rate of increase and decrease of *E* decreases and *E* reaches its maximum peak earlier and at a lower value. As *q* changes, there is no obvious pattern of change in *I*, but it can be concluded that *q* has a significant effect on the change in the value of *I*. Figures 5 and 6 illustrate that when *R*_0_ > 1, the changes of *q* and *δ* have significant effects on the trends of *S*, *E*, and *I*. And when *R*_0_ > 1, the endemic equilibrium point is tending to be stable.

### 3.2. Model Simulation

**Figure 7:**
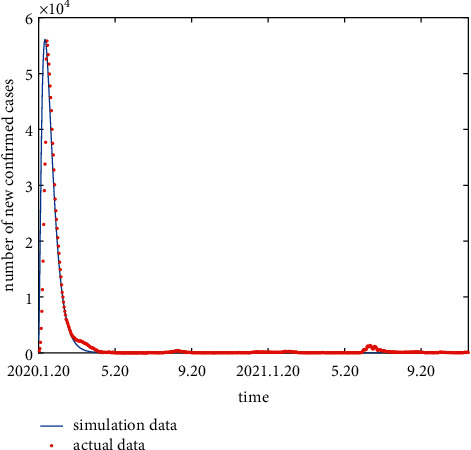
Model of the amount of confirmed cases in China per day.

The real data of China in this paper are from the official website of the WHO. We preprocess the data, remove some bad points, and make the time continuous so that the data can be better adapted to the SEIR-QV model. The Chinese COVID-19 outbreak is fitted based on the SEIR-QV model. The parameters used in the model are listed as follows: *c*=0.219, *η*=0.00714, *q*=0.5, *δ*=0.2, *β*=1 × 10^−8^, *υ*=0.275, *b*_1_=0.05, *σ*=1/7, *α*_1_=0.035, Λ=0.00334, *b*_2_=0.05, *α*_2_=0.035, and *r*=0.9493. The simulation results and the actual data are shown in Figure 7.

The Chinese government has decisively taken strict preventive and control measures following the emergence of the COVID-19 outbreak. These prevention and control measures, along with vigorous advocacy by the Chinese government, have reduced exposure rates *c* and increased isolation rates *q* and minimized the development of aggregated outbreaks. The COVID-19 outbreak has become manageable and stable. As can be seen in Figure 7, the simulated data fit better with the real data from January 20, 2020 to May 20, 2021. However, there is a small outbreak of the epidemic in China in June 2021. The main reason is that the epidemic is out of control in many places abroad, and people at home are taking it lightly and weakening the efforts of prevention and control, thus giving the opportunity for the epidemic to spread.

**Figure 8:**
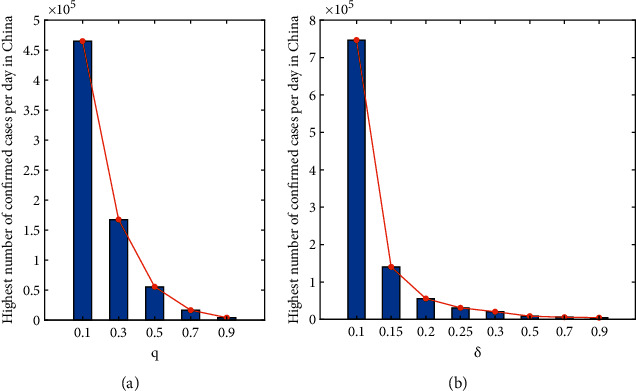
Impact of control strategies on the maximum number of daily confirmed cases of the epidemic in China: (a) *δ*=0.2 and (b) *q*=0.5.

As can be seen in Figure 8, both the isolation ratio and vaccine coverage play an important role in the control of the outbreak. The highest number of confirmed cases per day in China decreases as both *q* and *δ* increase.

The correlation coefficient is used to measure the accuracy of the fit, and the value of *R* is found to be 0.9394. Therefore, the results show that the SEIR-QV model can be used for COVID-19 epidemic development status assessment and has important implications.

## 4. Conclusion

An SEIR-QV model based on vaccination, isolation strategies, and the impact of different parameters on the development of infectious diseases is developed. The equilibrium point and stability of the new model are proved by using the basic regeneration number and the Lyapunov function theory. Simulation experiments show that the new method has certain theoretical value for analyzing and predicting the development of the COVID-19 epidemic. We can get the conclusion that there is a significant impact on the development of infectious diseases by different vaccination and isolation strategies.

## Figures and Tables

**Table 1:** Values of disease-free equilibrium points *q* and *δ*.

*q*	*δ*	*R*_0_
0.9	0.1	0.7289
0.9	0.5	0.1458
0.9	1	0.0729
0.1	1	0.6560
0.5	1	0.3645
0.9	1	0.5729

**Table 2:** Values of endemic equilibrium points *q* and *δ*.

*q*	*δ*	*R*_0_
0.1	0.05	13.12
0.1	0.1	6.5602
0.1	0.5	1.3120
0.1	0.05	13.12
0.5	0.05	7.2891
0.9	0.05	1.4578

## Data Availability

The dataset used to support the findings of this study is available from the corresponding author upon request.
